# Socio-economic disparities and returning to work following an injury

**DOI:** 10.1186/s13584-020-00392-3

**Published:** 2020-07-02

**Authors:** Bella Savitsky, Irina Radomislensky, Sharon Goldman, Natalia Gitelson, Zhanna Frid, Kobi Peleg

**Affiliations:** 1 Israel National Center for Trauma and Emergency Medicine Research, Gertner Institute for Epidemiology and Public Health Policy Research, Sheba Medical Center, Tel-Hashomer, 52621 Ramat Gan, Israel; 2 The National Insurance Institute of Israel, Research Center, Sderot Weizmann 13, Jerusalem, Israel; 3 Department of Disaster Management, School of Public Health, Tel Aviv University, Tel-Aviv, Israel

**Keywords:** Socio-economic status, Income, Ethnicity, Injury, Work absenteeism, Return to work, Out of work stay, Disability

## Abstract

**Background:**

Traumatic injury is one of the main reasons for temporary and permanent occupational disability.

The objective of this study was to define the role of socio-economic position on post-injury occupational absenteeism.

**Methods:**

This was a nationwide retrospective cohort study, based on linking The Israeli National Trauma Registry (INTR) and the National Insurance Institute (NII) databases.

The study population included 44,740 injured workers (residents of Israel, aged 21–67, hospitalized between 2008 and 2013 and employed prior to injury as salaried workers). Logistic-regression models tested the probability of not returning to work (RTW).

**Results:**

The majority of the study population (61%) RTW within 1 month following the injury event. Income prior to injury was significantly associated with longer out of work stay, explaining 9% variance. A significant interaction (*p* value < 0.0001) was found between age and income on out of work stay more than 1 month, 1 year and 2 years. Logistic regression models of out of work stay were conducted separately for all age groups. Lower income was associated with greater chance for out of work stay for more than 1 month; and the gap between the lowest and highest income quartiles was greater among older workers (age 55+), where there was an elevenfold increase in probability of not RTW among casualties from the lowest vs. highest income quartile. In comparison to other population groups, Arabs were at greater odds of longer out of work stay following an injury. Among injured persons recognized by the NII as having occupational injuries, the odds for not RTW within a month, a year and 2 years were respectively 3.9, 2.5 and 2.2 times significantly greater in comparison to employees injured outside the workplace.

**Conclusions:**

This study identified population groups with a high probability of not RTW following an injury requiring hospitalization. Intervention programs for injured employees should promote early rehabilitation and aim to shorten out of work stay. These programs should be ethnically adapted and focus on underprivileged and disadvantaged populations.

## Background

Participating in an occupational activity is a financial necessity for the majority of the population and the ability to work is associated with healthier physical and mental well-being, increased self-esteem, and community connectedness [1]. Traumatic injury is one of the main reasons for temporary or permanent occupational disability [2], which hinders a productive lifestyle and contributes to a financial burden both to the individual and the economy [3].

Socio-Economic Status (SES) has been found to be associated with work absenteeism [4, 5]. The length of work absenteeism is longer and the probability of not returning to occupational activity is greater [4, 6–9] among low income injured employees, physical labor workers, and those who noted insecure status in the workplace.

The population of Israel in 2018, included 8,847,400 citizens residing in the country, of which 4,067,700 comprised the working population [10]. It should be noted that Israeli citizens and residents, including new immigrants, are entitled to health insurance and various other health care and social services and benefits. The Israeli population is characterized by a unique ethnic composition: Jews born in Israel or residing in Israel most of their life; immigrants, mainly from the Former Soviet Union (FSU) and from Ethiopia; and Israeli Arabs [11]. Beginning in 1990, large waves of immigrants began arriving from the FSU and currently makeup 10% of the Israeli population and include 1,040,000 citizens [12]. Immigrants differ in their culture and language from non-immigrant Jews [11].

The Ethiopian community is another unique ethnic minority living in Israel, which includes approximately 85,000 Ethiopian born Israeli citizens [10]. While the majority of Ethiopian Jews immigrated to Israel in the 1980’s and 1990’s, immigration has continued at a smaller rate. This ethnic community has experienced many challenges in social integration and absorption into Israeli society [13]. In addition, a high incidence of violence related injuries requiring hospitalization have been reported among Ethiopian immigrants [14, 15].

Arabs comprise 21% of the Israeli population, or about 1.8 million citizens, and comprise 13% of the Israeli workforce [10]. The majority of the Arab population (84%) are Muslims. Arabs and Jews differ in religion, culture and language. The Arab population lives in mostly all-Arab communities located in rural areas in Northern and Southern Israel [16]. Arabs in Israel have a history of lower income, poorer education and greater unemployment [11]. These factors have contributed to a gap in health parameters and life expectancy between Jews and Arabs [17].

In Israel, health-related inequalities have been studied and reported in the professional literature [11, 18–22]. Ethnicity, religion, socioeconomic position, education, employment status and geographic location all play a part in health disparities and in widening the socioeconomic gap (as assessed by the Gini coefficient-a marker of income variance) [11, 18, 20].

Following an injury causing work absenteeism, any resident can request a stipend from the NII. After each case is evaluated, the individual may be approved for receiving an allowance for an “occupational injury” (which is calculated on the basis of salary) or a “non-occupational injury” (which is based on the average monthly salary in the economy).

A previous study proposed a model for predicting the duration of out of work stay following an injury related hospitalization [23]. The objective of this study was to define the role of SES on post-injury out of work stay, taking into account various factors including ethnicity and income characteristics of the injured. In addition, we hoped to be able to determine, on the basis of the data, ways that intervention programs might best prevent long out of work stay.

## Methods

This is a retrospective cohort study, based on linking two national databases: the Israeli National Trauma Registry (INTR) and the National Insurance Institute (NII).

The INTR provides comprehensive data on hospitalized trauma patients from all six Level I trauma centers (TC) and 14 of the 20 Level II TCs in Israel. Detailed information regarding data collection by the INTR and the NII and data linkage between these databases has been previously described [23].

### Study population

Among the majority of the Israeli Jewish population, higher education and employment begin following mandatory military service (age 21). In addition, retirement age for women is 62 and 67 for men. Thus, the inclusion criterion for this study was to be a resident of Israel, aged 21–67, injured and hospitalized between January 1, 2008 and December 31, 2013, who was employed prior to injury as a salaried worker. The status “worked prior to injury” was met if the injured person received a salary during the 2 months prior to the injury event. Persons who made suicide attempts, died during hospitalization or during the first month following the injury event, were unemployed prior to the injury event, or were self-employed workers, were excluded. Injured persons with missing information regarding ethnic group (*n* = 187, 0.4%) were excluded from the analysis. In addition, 364 (0.8%) of the injured were excluded, as they died during the 2 years following the injury event. The final study population includes 44,740 hospitalized casualties.

### Study variables

*Return to work* (RTW) was defined by time from the injury event to the month of first post-injury salary reported.

*Duration of out of work stay* was calculated as the difference between the date of the first post-injury salary and the date of the injury event. Out of work stay was categorized into three dichotomous variables (out of work stay more than 1 month; more than 1 year; more than 2 years).

Length of follow up was calculated using the termination of surveillance date (01.12.2014) or date of death and the date of the injury event.

*Age groups* were categorized: 21–34; 35–44; 45–54; 55+ with more than 5 years before retirement age; 55+ with less than 5 years before retirement age.

*Gender*: male/female.

*Family status* during the month of the injury event was used as a categorical variable: Married with children; Married without children; Single with children; Single without children.

*Population group*: Immigrants from Former Soviet Union (FSU) who immigrated from 1990 on ward; Ethiopian Immigrants (EI); Israeli Arabs (IA); Other Israelis (OI) (Jews excluding immigrants from FSU and EI).

*SES* was assessed using income in New Israeli Shekels, NIS. *Income* was based on the month prior to the injury and categorized using 25% percentiles.

*Previous Disability* was reported in the NII database and comprised a dummy variable: yes/no.

*Injury Mechanism* was categorized as: Burns; Road Traffic Accidents (RTA); Violence; Falls and Other unintentional injuries.

*Injury Severity Score (ISS)* - the sum of the squares of the single highest Abbreviated Injury Scale score for each of the three most severely injured body regions [24] categorized 1-8 (mild injury); 9-14 (moderate injury); 16-24 (severe injury) and 25+ (critical injury) [25].

*Injury Circumstances* were defined as: Injured at work-with reconition of the NII; Injured at work- without recognition of the NII; Not Injured at work.

The profession and job description of an injured individual is not included in the NII database and thus was not available in this study.

### Statistical analysis

A univariate analysis examined the association between each independent variable and out of work stay of one month, one year and two years using χ^2^ test. A multivariable analysis with logistic regression approach included all variables found in univariate analysis to be statistically associated with out of work stay. Before including independent variables in multivariable analysis, correlation between the variables were checked with Kendall's Tau coefficient. Most of the correlations were weak, with the strongest reaching Kendall's Tau coefficient of 0.4. Multicollinearity between all variables included in the multivariable analysis was assessed: maximum Variance Inflation Factors (VIF) was 2.4.

Separate models were constructed with three dependent variables:1) Out of work stay more than one month, 2) Out of work stay more than one year, and 3) Out of work stay more than two years. Out of work stay for more than two years was investigated among hospitalized workers injured between 2008-2012 (*n* = 36,504, excluding 8,236 injured in 2013 and thus, could not complete the two-year follow-up).

Using the R package *margins* [26] average marginal effects (AME) of the regression models were calculated and are presented in the Tables 9-14 in Appendix.

In addition, interactions between SES and other variables were tested. Significant interactions (*p* value < 0.0001) between age and income, regarding out of work stay for more than 1 month, 1 year and 2 years, were found. Thus, logistic regression models of out of work stay were conducted separately for each age group.

Analyses were carried out with SAS V.9.2 statistical software, with SPSS version 22.0 statistical package and with R package version 0.3.23. For all analyses performed, a value of *p* < 0.05 was considered statistically significant. C-statistic was calculated to assess the predictive ability of the logistic regression models, a value above 0.7 indicated good predictive ability of the model [27].

## Results

### Population characteristics

During the study period, 44,740 persons having injuries were hospitalized. The mean age was 38.7 years and almost half (46%) of the hospitalizations were among the 21-34 yearage group (Table 1). Men comprised almost 70% of all the hospitalized casualties, and 67.8% of those with occupational injuries. The proportion of Arabs (25%) was much higher than their proportion in the work force (13%) [10]. Immigrants from the FSU accounted for 14% of the study population (while they account for 10% of the general population), EI accounted for 1.5% and the remaining 59% included OI.

**Table 1:** Study population demographic and injury characteristics by ethnic group

Demographic, Socioeconomic and Injury Characteristics	Other Israelis	Immigrants from FSU	Ethiopian Immigrants	Israeli Arabs	Total *n* = 44,740
	*n* = 26,652 59.6%	*n* = 6,241 13.9%	*n* = 686 1.5%	*n* = 11,161 24.9%	
Age (years), mean (SD)	40.2 (13.3)	41.2 (13.0)	35.4 (11.2)	33.9 (10.5)	38.7 (12.9)
**Age (years), (%)**					
21–34	42.5	36.9	54.7	58.8	46.0
35–44	19.9	22.1	24.1	23.6	21.2
45–54	17.6	20.7	13.7	12.7	16.7
55+ with more than 5 years before retirementage	8.6	10.0	3.8	3.1	7.4
55+ with less than 5 years before retirement age	11.4	10.4	3.8	1.7	8.7
**Gender (%)**					
Male	59.3	66.5	73.5	82.6	66.3
Female	40.7	33.5	26.5	17.4	33.7
**Family Status** (%)					
Married with children	44.1	28.7	44.6	56.8	45.1
Married without children	20.4	28.8	5.2	11.1	19.0
Single with children	5.8	10.0	11.7	1.3	5.3
Single without children	29.8	32.5	38.5	30.7	30.5
**Income (NIS)**					
Mean (SD)	9,583 (10,265)	6,970 (5,440)	5,145 (3,056)	5,840 (4,222)	8,217 (8,624)
Median [IQR]	6,678 [4,116; 11,518]	5,863 [3,948;8,419]	4,826 [3,221;6,533]	4,885 [3,600;7,023]	5,930 [3,893;9,506]
**Income Quartile (%)**					
Highest income (4th Quartile)	32.7	18.6	5.8	11.4	25.0
3th Quartile	23.8	30.7	27.1	24.6	25.0
2nd Quartile	20.8	26.3	32.2	33.9	25.0
Lowest income (1st Quartile)	22.6	24.5	34.8	30.1	25.0
**Previous Disability (%)**	2.3	1.9	1.9	1.1	1.9
**Injury Mechanism (%)**					
Burns	2.1	2.8	2.2	2.9	2.4
Road Traffic Accidents (RTA)	41.5	29.1	30.9	28.3	36.3
Violence	3.5	10.5	23.2	11.3	6.7
Falls	34.9	31.2	19.8	27.4	32.3
Other non-intentional^a^	17.9	26.4	23.9	30.0	22.2
**Injury Severity (ISS)**^b^ (%)					
Mild (ISS 1–8)	74.5	74.3	75.7	78.0	75.4
Moderate (ISS 9–14)	17.8	17.1	13.7	13.7	16.6
Severe (ISS 16–24)	4.9	5.4	7.3	4.9	5.0
Critical (ISS 25+)	2.8	3.2	3.4	3.4	3.0
**Injury Circumstances (%)**					
Injured not at work	61.1	52.7	56.9	49.5	57.0
Injured at work - without recognition of NII	5.8	5.3	3.4	9.2	6.5
Injured at work - with recognition of NII	33.1	42.0	39.8	41.4	36.5
**Previously Disability (%)**	2.3	1.9	1.9	1.1	1.9

^a^Other unintentional injuries include injuries from objects and people that occurred without any intention of causing damage to oneself or others

^b^*ISS* Injury Severity Score

Road accidents and falls were the cause for 36 and 32% of the hospitalizations, respectively. The majority (75%) of hospitalized patients sustained minor injuries (ISS 1–8).

Among the 43% of injured workers, an event caused injury occurred at the workplace. Among them, 85% were recognized as having an “occupational injury” by the NII, while 15% were not granted recognition.

### Out of work stay (univariate analysis)

The median time span of absence from work was 1 month (Inter Quarter Range: 1–4 months).

The majority of the study population (61%) RTW within 1 month from the injury event (39% stayed out of work for more than a month), 12% did not RTW during the first year following the injury, 8% did not RTW during the 2 years following the injury event (out of those hospitalized between 2008 and 2012) and 6% did not RTW during the entire study period (Table 2).

**Table 2:** The univariate analysis of % of not RTW within 1 month, 1 year and 2 years, by demographic and injury characteristics

Demographic and injury Characteristics	Non-return to work (%)		
	within 1 month	within 1 year	within 2 years^**a**^
	39%	12%	8%
**Age**			
21–34	42.9	11.8	7.2
35–44	38.1	12.8	8.0
45–54	34.6	12.5	7.9
55+ with more than 5 years before retirement age	33.3	10.9	7.8
55+ with less than 5 years before retirement age	30.3	10.3	8.1
*p* value for χ^2^ test	< 0.0001	< 0.0001	0.134
**Gender**			
Male	44.1	14.5	9.4
Female	28.0	6.8	4.7
*p* value for χ^2^ test	< 0.0001	< 0.0001	< 0.0001
**Family Status**			
Married with children	36.6	11.5	7.4
Married without children	32.7	10.6	7.0
Single with children	38.4	13.6	8.3
Single without children	45.5	13.0	8.2
*p* value for χ^2^ test	< 0.0001	< 0.0001	0.011
**Population Group**			
Other Israelis	32.5	8.8	5.7
Immigrants from FSU	38.9	10.0	6.0
Ethiopian Immigrants	43.0	14.3	9.6
Israeli Arabs	53.0	20.2	13.8
*p* value for χ^2^ test	< 0.0001	< 0.0001	< 0.0001
**Income**			
Highest income (4^th^Quartile)	16.9	3.6	2.2
3^th^Quartile	34.9	8.9	5.3
2^nd^Quartile	47.4	15.4	9.6
Lowest income (1^st^Quartile)	55.5	19.8	13.7
*p* value for χ^2^ test	< 0.0001	< 0.0001	< 0.0001
**Previous Disability**			
Yes	49.6	20.0	17.5
No	38.5	11.8	7.6
*p* value for χ^2^ test	< 0.0001	< 0.0001	< 0.0001
**Injury Characteristics**			
**Injury Mechanism**			
Burns	29.4	7.7	4.9
RTA	39.1	11.5	7.7
Violence	34.5	11.7	7.4
Falls	36.6	12.4	8.3
Other unintentional	43.3	12.5	7.7
*p* value for χ^2^ test	< 0.0001	< 0.0001	0.003
**Injury Severity (ISS)**^**b**^			
Mild (ISS 1–8)	36.3	9.5	5.7
Moderate (ISS9–14)	43.0	15.8	10.6
Severe (ISS 16–24)	48.8	19.9	14.4
Critical (ISS 25+)	59.0	39.0	34.0
*p* value for χ^2^ test	< 0.0001	< 0.0001	< 0.0001
**Injury Circumstances**			
Injured not at work	28.5	8.2	5.5
Injured at work - without recognition of NII	24.6	7.4	5.0
Injured at work - with recognition of NII	57.0	18.6	11.9
*p* value for χ^2^ test	< 0.0001	< 0.0001	< 0.0001

^a^Refers to those injured between 2008–2012 (*n* = 36,504)

^b^*ISS* Injury Severity Score

### Out of work stay (multivariate analysis)

The Model which predicted not-RTW within 1 month achieved C-statistic of 0.78 and models predicting not RTW within 1 and 2 years achieved C-statistic of 0.79.

Table 3 describes the multivariate logistic regression model for predicting not RTW within 1 month, one-year and two-years. In addition to income, the analysis was adjusted for age, gender, population group, family status, injury circumstances, previous disability, injury mechanism and injury severity.

**Table 3:** The multivariate logistic regression model^a^ for predicting not RTW within 1 month, 1 year and 2 years, by demographic and injury characteristics

Demographic and injury characteristics	Odds Ratio (OR) [Confidence Interval (95%)]		
	Not RTW within 1 month (39%)	Not RTWwithin 1 year (12%)	Not RTWwithin 2 years^**b**^ (8%)
**Age**			
21–34	**1.329 [1.208–1.463]**	**0.750 [0.653–0.861]**	**0.537 [0.450–0.642]**
35–44	**1.278 [1.15–1.419]**	0.969 [0.835–1.125]	**0.690 [0.569–0.837]**
45–54	**1.116 [1.008–1.235]**	1.046 [0.907–1.208]	**0.786 [0.653–0.947]**
55+ with more than 5 years before retirement age	0.953 [0.850–1.068]	0.851 [0.723–1.001]	**0.779 [0.633–0.958]**
55+ with less than 5 years before retirement age	1.0^c^	1.0^c^	1.0^c^
**Gender** (male vs. female)	**1.947 [1.846–2.053]**	**2.038 [1.878–2.211]**	**1.813 [1.626–2.022]**
**Family Status**			
Married with children	**1.086 [1.009–1.168]**	1.054 [0.949–1.169]	**1.154 [1.004–1.327]**
Married without children	1.0^c^	1.0^c^	1.0^c^
Single with children	**1.273 [1.136–1.426]**	**1.449 [1.239–1.695]**	**1.511 [1.222–1.868]**
Single without children	**1.180 [1.096–1.271]**	1.011 [0.909–1.124]	1.081 [0.938–1.245]
**Population Group**			
Other Israelis	1.0^c^	1.0^c^	1.0^c^
Immigrants from FSU	**1.069 [1.002–1.140]**	0.913 [0.827–1.008]	**0.843 [0.735–0.967]**
Ethiopian Immigrants	0.960 [0.811–1.136]	1.216 [0.968–1.528]	1.252 [0.921–1.702]
Israeli Arabs	**1.592 [1.508–1.680]**	**1.932 [1.797–2.078]**	**2.111 [1.916–2.326]**
**Income**			
Higher income (4^th^Quartile)	1.0^c^	1.0^c^	1.0^c^
3^th^Quartile	**2.401 [2.241–2.571]**	**2.414 [2.136–2.728]**	**2.369 [1.990–2.819]**
2^nd^Quartile	**4.433 [4.135–4.754]**	**4.785 [4.254–5.384]**	**4.527 [3.835–5.343]**
Lower income (1^st^Quartile)	**8.165 [7.594–8.870]**	**8.117 [7.216–9.131]**	**8.005 [6.795–9.431]**
**Injury Circumstances**			
Injured not at work	1.0^c^	1.0^c^	1.0^c^
Injured at work - without recognition of NII	**0.746 [0.677–0.822]**	**0.822 [0.705–0.958]**	0.860 [0.700–1.057]
Injured at work - with recognition of NII	**3.872 [3.686–4.068]**	**2.483 [2.319–2.658]**	**2.220 [2.028–2.431]**

^a^The model is adjusted for all variables presented in the Table and in addition to injury mechanism, injury severity and prior-to injury disability

^b^Refers to those injured between 2008–2012

^c^Reference group (OR = 1.0)

Low SES, based on income, was associated with a significantly higher probability of not RTW within a month, 1 and 2 years, while a dose-response relationship between income and the odds of not RTW was observed. In comparison to casualties in the highest income level, the probability of not RTW was more than eight times greater among those in the lowest income bracket (OR = 8.17; 95% CI:7.59–8.87), more than four-fold greater among casualties in the second quartile (OR = 4.43; 95% CI: 4.14–4.75) and more than twofold greater among injured persons with income level in the third quartile (OR = 2.40; 95% CI 2.24–2.57). According to the results of the marginal effects, injured persons from the lowest (first) income quartile were 38.9% (95% CI: 37.7–40.1%) (Table 9 in Appendix) more likely to not RTW within a month in comparison with those from the highest income quartile. Injured persons from the second and the third income quartiles (27.6% [CI 95%: 26.4–28.8%] and 16.2% [15.0–17.5% respectively]) were more likely to not RTW within a month compared to casualties from the highest income quartile (Table 9 in Appendix).

For immigrants from the FSU, immigrants from Ethiopia, and other Israeli Jews the probability of not RTW within 1 month was similar. In contrast, among Arabs the odds of not RTW were almost 60% higher (OR = 1.59; 95% CI 1.51–1.68) in comparison to Israeli Jews (marginal effect of 8.6% [CI 95%: 7.6–9.6%]) (Table 9 in Appendix). As absence from work extended, the gap expanded; for example, in comparison to other population groups, among Arabs the probability of not RTW within a year was 93% greater (OR = 1.93; 95% CI 1.79–2.08) and not RTW within 2 years was more than double (OR = 2.11; 95% CI 1.92–2.33).

Among persons injured at the workplace and recognized by the NII as having “occupational injuries”, the odds for not RTW within a month, a year and 2 years were respectively 3.9, 2.5 and 2 times significantly greater, in comparison to casualties not injured at work or not recognized by the NII.

The study population was stratified by age following the statistically significant interaction (*p* value < 0.0001) between age and income. In addition, a separate logistic regression model, with all previously mentioned independent variables, was conducted for each age group (Tables 4, 5, 6, 7, 8). For eachage group the probability of not RTWwas greater among those with lower income, with a dose-response relationship between income and probability of not RTW.

**Table 4:** The multivariate logistic regression model^a^ for predicting not RTW within 1 month, 1 year and 2 years, by demographic and injury characteristics among aged 21–34

Demographic and injury characteristics	Odds Ratio (OR) [Confidence Interval (95%)]		
	Not RTW within 1 month	Not RTWwithin 1 year	Not RTWwithin 2 years^**b**^
**Gender** (male vs. female)	**1.842 [1.704–1.992]**	**2.112 [1.855–2.404]**	**2.005 [1.675–2.399]**
**Family Status**			
Married with children	**1.154 [1.016–1.311]**	1.016 [0.842–1.226]	**1.135 [0.876–1.471]**
Married without children	1.0^c^	1.0^c^	1.0^c^
Single with children	**1.579 [1.282–1.946]**	**1.634 [1.216–2.196]**	**1.622 [1.082–2.430]**
Single without children	**1.305 [1.150–1.480]**	0.908 [0.755–1.091]	0.955 [0.740–1.232]
**Population Group**			
Other Israelis	1.0^c^	1.0^c^	1.0^c^
Immigrants from FSU	0.980 [0.885–1.085]	0.951 [0.805–1.123]	0.907 [0.718–1.147]
Ethiopian Immigrants	0.959 [0.766–1.202]	1.348 [0.983–1.849]	1.387 [0.900–2.139]
Israeli Arabs	**1.481 [1.380–1.590]**	**1.875 [1.693–2.076]**	**2.016 [1.756–2.315]**
**Income**			
Higher income (4^th^Quartile)	1.0^c^	1.0^c^	1.0^c^
3^th^Quartile	**1.758 [1.565–1.974]**	**1.608 [1.294–1.999]**	**1.466 [1.080–1.989]**
2^nd^Quartile	**3.065 [2.735–3.434]**	**2.943 [2.391–3.622]**	**2.648 [1.983–3.535]**
Lower income (1^st^Quartile)	**5.253 [4.679–5.896]**	**4.610 [3.749–5.672]**	**4.188 [3.144–5.579]**
**Injury Circumstances**			
Injured not at work	1.0^c^	1.0^c^	1.0^c^
Injured at work - without recognition of NII	**0.638 [0.558–0.730]**	**0.652 [0.513–0.830]**	0.795 [0.580–1.089]
Injured at work - with recognition of NII	**3.251 [3.025–3.494]**	**2.311 [2.090–2.556]**	**2.168 [1.891–2.485]**

^a^The model is adjusted for all variables presented in the Table and in addition to injury mechanism, injury severity and prior-to injury disability

^b^Refers to those injured between 2008–2012

^c^Reference group (OR = 1.0)

**Table 5:** The multivariate logistic regression model^a^ for predicting not RTW within 1 month, 1 year and 2 years, by demographic and injury characteristics among aged 35–44

Demographic and injury characteristics	Odds Ratio (OR) [Confidence Interval (95%)]		
	Not RTW within 1 month	Not RTWwithin 1 year	Not RTWwithin 2 years^**b**^
**Gender** (male vs. female)	**2.078 [1.842–2.343]**	**2.295 [1.914–2.751]**	**1.957 [1.540–2.487]**
**Family Status**			
Married with children	0.989 [0.773–1.265]	1.032 [0.740–1.439]	0.883 [0.571–1.364]
Married without children	1.0^c^	1.0^c^	1.0^c^
Single with children	1.126 [0.850–1.491]	1.441 [0.987–2.103]	1.165 [0.706–1.924]
Single without children	1.091 [0.827–1.438]	0.988 [0.680–1.436]	0.857 [0.525–1.398]
**Population Group**			
Other Israelis	1.0^c^	1.0^c^	1.0^c^
Immigrants from FSU	0.958 [0.830–1.106]	0.824 [0.665–1.021]	**0.701 [0.517–0.951]**
Ethiopian Immigrants	0.909 [0.641–1.289]	1.232 [0.785–1.933]	1.399 [0.772–2.532]
Israeli Arabs	**1.720 [1.529–1.934]**	**1.772 [1.518–2.068]**	**1.804 [1.468–2.218]**
**Income**			
Higher income (4^th^Quartile)	1.0^c^	1.0^c^	1.0^c^
3^th^Quartile	**2.669 [2.332–3.054]**	**2.851 [2.246–3.619]**	**3.077 [2.145–4.412]**
2^nd^Quartile	**4.941 [4.283–5.702]**	**5.778 [4.578–7.294]**	**5.918 [4.175–8.389]**
Lower income (1^st^Quartile)	**9.183 [7.841–10.754]**	**10.142 [7.995–12.867]**	**11.766 [8.307–16.667]**
**Injury Circumstances**			
Injured not at work	1.0^c^	1.0^c^	1.0^c^
Injured at work - without recognition of NII	**0.767** **0.624–0.941**	0.859 [0.632–1.167]	0.894 [0.591–1.348]
Injured at work - with recognition of NII	**4.142 [3.719–4.612]**	**2.471 [2.13092.868]**	**2.106 [1.725–2.570]**

^a^The model is adjusted for all variables presented in the Table and in addition to injury mechanism, injury severity and prior-to injury disability

^b^Refers to those injured between 2008–2012

^c^Reference group (OR = 1.0)

**Table 6:** The multivariate logistic regression model^a^ for predicting not RTW within 1 month, 1 year and 2 years, by demographic and injury characteristics among aged 45–54

Demographic and injury characteristics	Odds Ratio (OR) [Confidence Interval (95%)]		
	Not RTW within 1 month	Not RTWwithin 1 year	Not RTWwithin 2 years^**b**^
**Gender** (male vs. female)	**2.329 [2.021–2.683]**	**2.430 [1.990–2.967]**	**1.935 [1.491–2.512]**
**Family Status**			
Married with children	1.093 [0.946–1.263]	0.950 [0.779–1.159]	1.114 [0.850–1.461]
Married without children	1.0^c^	1.0^c^	1.0^c^
Single with children	1.244 [0.992–1.560]	1.161 [0.858–1.572]	**1.559 [1.047–2.321]**
Single without children	1.040 [0.858–1.261]	1.083 [0.836–1.405]	1.178 [0.829–1.673]
**Population Group**			
Other Israelis	1.0^c^	1.0^c^	1.0^c^
Immigrants from FSU	1.133 [0.969–1.324]	**0.793 [0.630–0.998]**	**0.786 [0.571–1.082]**
Ethiopian Immigrants	1.164 [0.726–1.865]	0.868 [0.465–1.619]	1.022 [0.475–2.200]
Israeli Arabs	**1.791 [1.539–2.085]**	**2.112 [1.756–2.541]**	**2.450 [1.925–3.117]**
**Income**			
Higher income (4^th^Quartile)	1.0^c^	1.0^c^	1.0^c^
3^th^Quartile	**2.389 [2.046–2.788]**	**2.718 [2.095–3.525]**	**2.924 [2.008–4.257]**
2^nd^Quartile	**5.518 [4.674–6.515]**	**5.782 [4.481–7.460]**	**5.737 [3.986–8.258]**
Lower income (1^st^Quartile)	**12.573 [10.479–15.087]**	**11.792 [9.125–15.240]**	**10.336 [7.217–14.803]**
**Injury Circumstances**			
Injured not at work	1.0^c^	1.0^c^	1.0^c^
Injured at work - without recognition of NII	0.883 [0.683–1.142]	1.172 [0.823–1.669]	1.091 [0.671–1.774]
Injured at work - with recognition of NII	**4.908 [4.325–5.569]**	**3.424 [2.867–4.090]**	**3.014 [2.380–3.817]**

^a^The model is adjusted for all variables presented in the Table and in addition to injury mechanism, injury severity and prior-to injury disability

^b^Refers to those injured between 2008 and 2012

^c^Reference group (OR = 1.0)

**Table 7:** The multivariate logistic regression model^a^ for predicting not RTW within 1 month, 1 year and 2 years, by demographic and injury characteristics among aged 55+ with more than 5 years before retirement age

Demographic and injury characteristics	Odds Ratio (OR) [Confidence Interval (95%)]		
	Not RTW within 1 month	Not RTWwithin 1 year	Not RTWwithin 2 years^**b**^
**Gender** (male vs. female)	**1.803 [1.391–2.337]**	**1.905 [1.295–2.803]**	**2.249 [1.338–3.783]**
**Family Status**			
Married with children	0.807 [0.637–1.021]	0.906 [0.654–1.256]	1.110 [0.746–1.650]
Married without children	1.0^c^	1.0^c^	1.0^c^
Single with children	0.987 [0.612–1.592]	1.220 [0.647–2.300]	0.506 [0.149–1.715]
Single without children	0.816 [0.640–1.040]	1.101 [0.787–1.539]	1.267 [0.830–1.934]
**Population Group**			
Other Israelis	1.0^c^	1.0^c^	1.0^c^
Immigrant from FSU	1.182 [0.955–1.463]	0.951 [0.698–1.295]	0.933 [0.623–1.399]
Ethiopian Immigrants	0.842 [0.346–2.047]	0.559 [0.125–2.508]	no participants
Israeli Arabs	**1.658 [1.260–2.182]**	**2.212 [1.590–3.078]**	**2.652 [1.767–3.982]**
**Income**			
Higher income (4^th^Quartile)	1.0^c^	1.0^c^	1.0^c^
3^th^Quartile	**2.566 [2.040–3.228]**	**2.195 [1.464–3.292]**	**1.810 [1.048–3.124]**
2^nd^Quartile	**5.096 [4.008–6.480]**	**6.107 [4.199–8.881]**	**4.482 [2.736–7.341]**
Lower income (1^st^Quartile)	**10.110 [7.679–13.311]**	**10.535 [7.131–15.563]**	**10.873 [6.680–17.698]**
**Injury Circumstances**			
Injured not at work	1.0^c^	1.0^c^	1.0^c^
Injured at work - without recognition of NII	**1.538 [1.050–2.253]**	1.477 [0.852–2.562]	1.074 [0.500–2.306]
Injured at work - with recognition of NII	**5.768 [4.748–7.008]**	**3.317 [2.520–4.367]**	**2.636 [1.871–3.715]**

^a^The model is adjusted for all variables presented in the Table and in addition to injury mechanism, injury severity and prior-to injury disability

^b^Refers to those injured between 2008–2012

^c^Reference group (OR = 1.0)

**Table 8:** The multivariate logistic regression model^a^ for predicting not RTW within 1 month, 1 year and 2 years, by demographic and injury characteristics among aged 55+ with less than 5 years before retirement age

Demographic and injury characteristics	Odds Ratio (OR) [Confidence Interval (95%)]		
	Not RTW within 1 month	Not RTWwithin 1 year	Not RTWwithin 2 years^**b**^
**Gender** (male vs. female)	**1.798 [1.505–2.148]**	**1.450 [1.139–1.845]**	1.201 [0.880–1.641]
**Family Status**			
Married with children	1.127 [0.718–1.770]	0.890 [0.487–1.625]	0.819 [0.380–1.764]
Married without children	1.0^c^	1.0^c^	1.0^c^
Single with children	1.934 [0.806–4.640]	0.802 [0.183–3.511]	0.594 [0.078–4.535]
Single without children	**1.437 [1.203–1.717]**	1.230 [0.965–1.567]	1.191 [0.877–1.618]
**Population Group**			
Other Israelis	1.0^c^	1.0^c^	1.0^c^
Immigrants from FSU	1.207 [0.968–1.475]	1.010 [0.764–1.333]	0.911 [0.633–1.310]
Ethiopian Immigrants	0.413 [0.162–1.055]	1.785 [0.641–4.973]	1.780 [0.442–7.172]
Israeli Arabs	**2.041 [1.450–2.874]**	**2.547 [1.725–3.760]**	**3.210 [1.999–5.156]**
**Income**			
Higher income (4^th^Quartile)	1.0^c^	1.0^c^	1.0^c^
3^th^Quartile	**2.995 [2.325–3.858]**	**1.958 [1.301–2.946]**	**2.048 [1.186–3.535]**
2^nd^Quartile	**5.420 [4.215–6.971]**	**3.586 [2.452–5.245]**	**3.839 [2.322–6.347]**
Lower income (1^st^Quartile)	**11.534 [9.050–14.699]**	**7.079 [4.997–10.029]**	**7.297 [4.600–11.576]**
**Injury Circumstances**			
Injured not at work	1.0^c^	1.0^c^	1.0^c^
Injured at work - without recognition of NII	0.898 [0.561–1.439]	0.729 [0.367–1.448]	0.582 [0.225–1.504]
Injured at work - with recognition of NII	**4.112 [3.471–4.870]**	**1.592 [1.272–1.992]**	**1.374 [1.036–1.822]**

^a^The model is adjusted for all variables presented in the Table and in addition to injury mechanism, injury severity and prior-to injury disability

^b^Refers to those injured between 2008–2012

^c^Reference group (OR = 1.0)

**Fig. 1:**
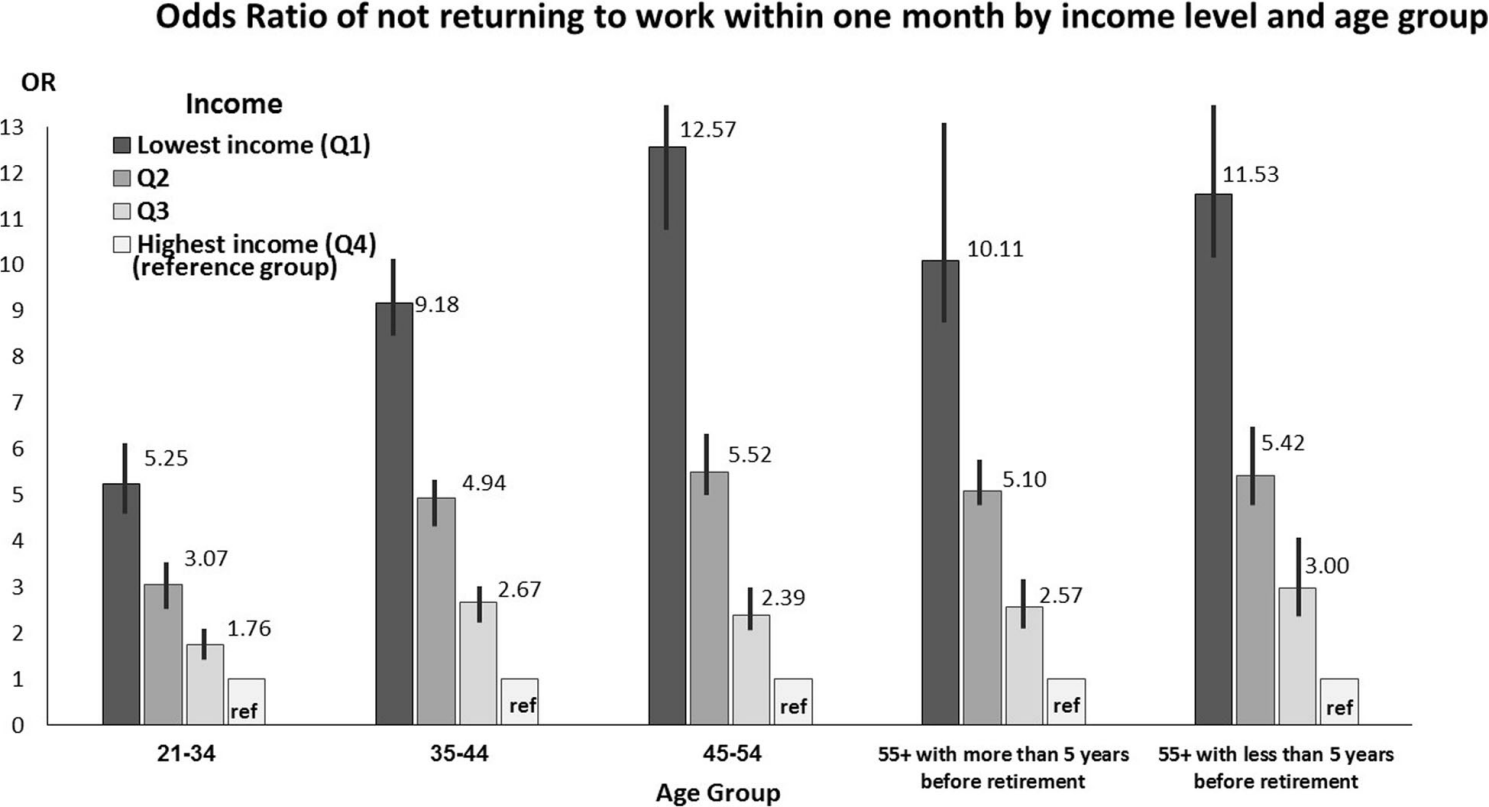
Odds Ratio of not returning to work within 1 month by income level and age group

Figure 1 depicts the comparison of OR for not RTW within 1 month, by age group and income level. Among young adults (aged 21–34 years), casualties in the highest income quartile, in comparison to casualties in the lowest income quartile, had a fivefold greater probability of not RTW within a month. The gap in probability of not RTW between the lowest and the highest income quartiles increased with age; among ages 35–44 a nine fold increase in OR was found, a twelvefold increase was reported among ages 45–54, and a tenfold and eleven-fold increase among ages 55 + .

**Fig. 2:**
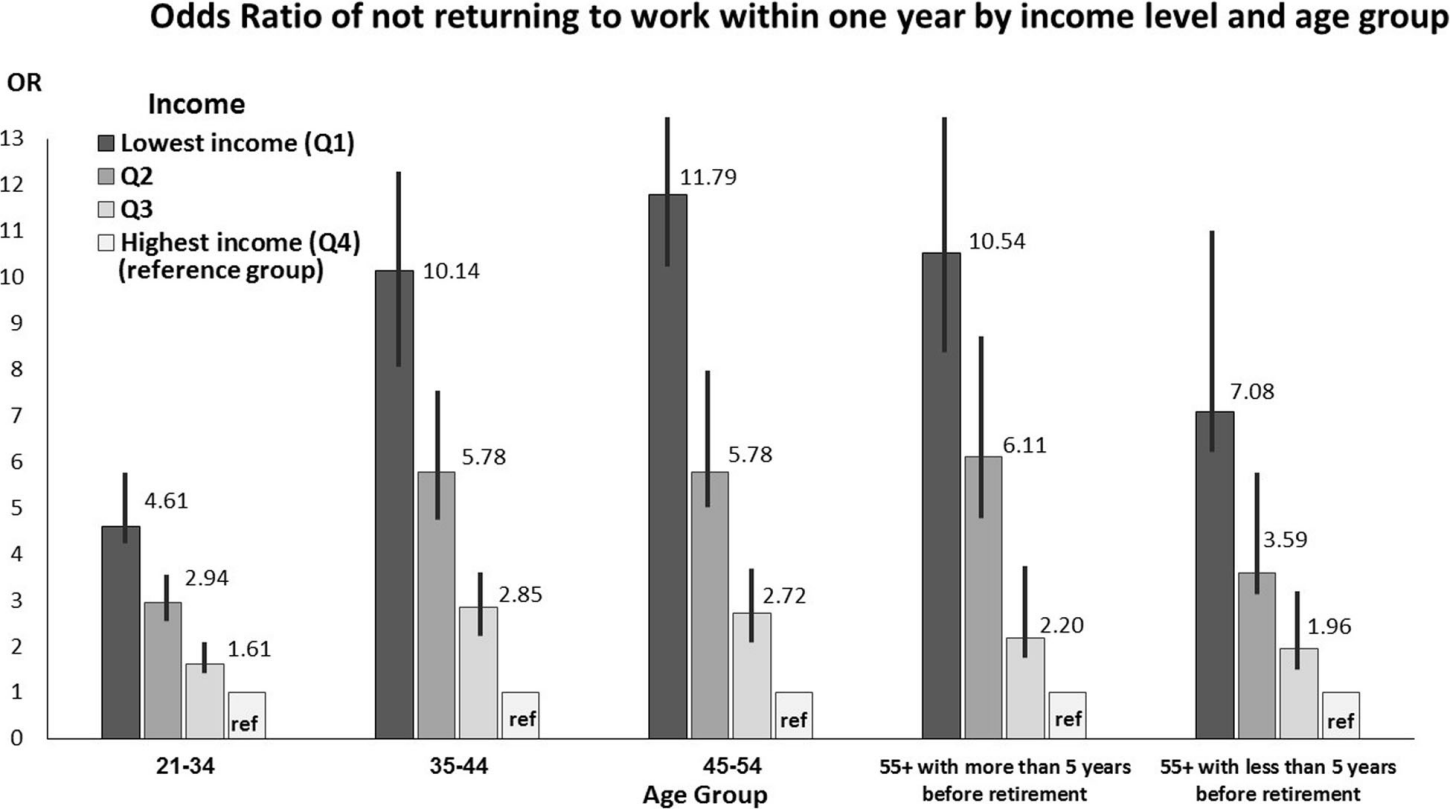
Odds Ratio of not returning to work within 1 year by income level and age group

**Fig. 3:**
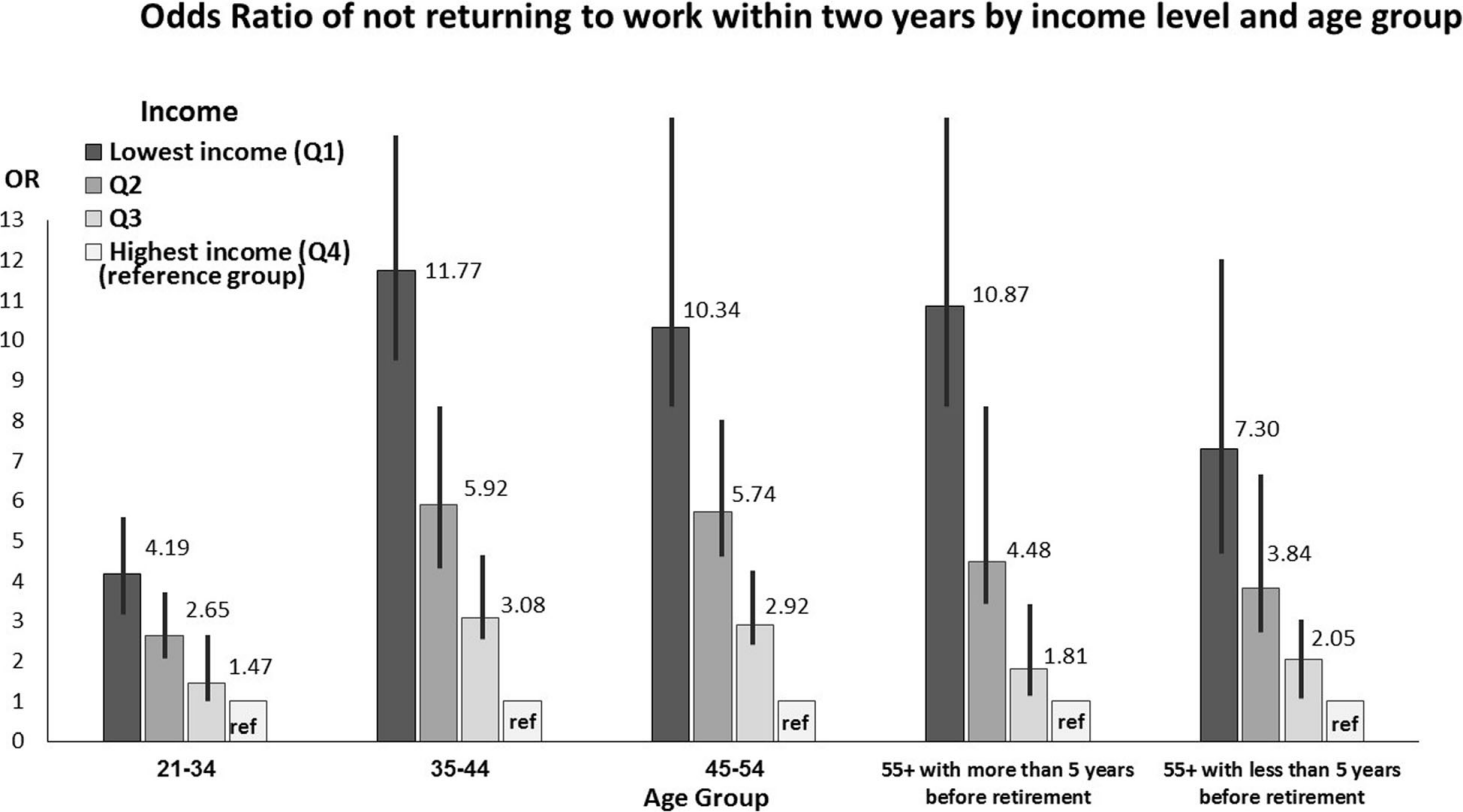
Odds Ratio of not returning to work within 2 years by income level and age group

For those injured workers who were close to retirement age, the gap between the highest and the lowest income levels decreased regarding the OR for not RTW within 1 year and 2 years (Figs. 2 and 3).

Income contributed significantly to the prediction of not RTW, and explained a 9% variance in the probability of not RTW within a month, and a 6% variance in the probability of not RTW within 1 and 2 years.

## Discussion

This study, which is based on national longitudinal data, is the first of its kind in Israel to investigate the impact of SES on post-injury out of work stay. In our study most of participants (61%) RTW within 1 month and 88% RTW within 1 year. Similar findings were described by researchers of an Australian study and an East China study. The Australian study reported that 68% of hospitalized injured patients RTW within 6 months following an injury event [28] while the study from East China claimed that 78% RTW within 8 months [29]. The results of the current study showed that income is significantly associated with RTW, that is, as income decreased the probability of not RTW increased. Likewise, an association between lower income and lower chance of RTW has been described in the professional literature [7, 9, 30–32]. In addition, the disparity between the lowest and the highest income quartile, regarding the probability of not RTW, was most notable amongst older workers. A literature search did not find any study which investigated an interaction between age and income in relation to RTW.

Our study ascertained that, in comparison to younger employees, older individuals had a greater probability of RTW within a month and a lower probability of RTW within 1 and 2 years. These findings coincide with outcomes from a study of 60,000 workers suffering from occupational injuries in the United States [33]. The study reported that for short term absence, older workers RTW sooner than younger workers. Another study, which focused on orthopedic trauma, also found that persons ages 45 and older injured in the non-workers’compensation group, RTW earlier than younger workers (among casualties in the workers’ compensation group, age was not associated with time until RTW) [34].

The fact that older workers are more likely to RTW within a month may be explained by an increased motivation to remain in the employment cycle, since finding a new job at a later time might be more challenging for an older worker. In addition, older personnel may occupy more administrative positions, which do not involve physical labor and therefore can RTW sooner.

The increased probability that older employees will not RTW within 1 and 2 years may be explained in part by slower recovery following an injury, and may reflect a possible obstacle in maintaining employment stability following long absence from work [4]. Another explanation may be early retirement options (employees in Israel who are insured under a budgetary pension arrangement are often entitled to early retirement). The odds of not RTW within 1 month and 1 year were similar for adults, ages 55+, with less than 5 years until retirement age and more than 5 years until retirement, however, the odds of not RTW within 2 years among those near retirement age were significantly greater. This finding supports the hypothesis that as retirement age nears, the likelihood of RTW decreases and the transition to retirement increases.

For all age groups, the probability of RTW depends on income (lower income coincides with an increased probability for longer out of work stay). However, among older employees, a huge gap between the lowest and the highest income brackets was observed. One explanation for the enormous differences between those with the lowest and highest income quartiles may be explained by job characteristics; for example, low income employees often have physical labor jobs, and thus RTW after being injured is often a difficult mission, and even more challenging among the older population.

Income was found to be an important predictor of RTW, with a dose-response association between income and RTW; the lower the income of the injured, the greater the chance of not RTW. Thus, absenteeism may be partially explained by the financial situation of the injured, which is supported by previous studies [4, 5, 32] showing that low income is a risk factor for longer out of work stay and a dose-response association exists between pre-injury income and RTW [7].

One reason for longer recovery periods among low income casualties may be due to rehabilitation costs, if they are not covered by health insurance or the NII. In addition, in the case of low salaried employees, following an injury the allowance received by the NII is often similar to the salary received prior to injury (the monthly compensation is based on mean salary in the economy) and thus, without RTW their standard of living remains stable.

Time until RTW was found to be associated not only to income, but to overall SES, which has also been reported literature [35–37]. Although compensation due to an occupational injury does not preclude the possibility of earning additional income, those injured in the work setting have longer out-of-work stays independent from injury characteristics [23]. In Israel, the monthly allowance, in the case of an occupational injury, is calculated on the basis of salary and is usually higher than a monthly allowance which is provided in the case of non-occupational injuries (which is based on the average monthly salary in the economy). A higher allowance (in case of an occupational injury) may serve as negative incentive for RTW.

Our results support the findings regarding the importance of family support in the case of injury. A previous study showed that post-injury rehabilitation was quicker among the those reporting strong social relationships [1]. Family support may serve as a facilitator for RTW, either due to practical assistance (e.g., driving the injured worker to work or appointments) or to emotional support [38]. In the current study, the probability of married workers (with and without children) to not RTW was lower in comparison to single employees. The highest odds of not RTW were observed among single employees with children. A possible explanation is that, in comparison to married individuals, single parents often have additional challenges in fulfilling parenting responsibilities and thus RTW following an injury is more challenging and may take longer [39].

The outcomes of this study reported disparities between the Jewish majority and the Arab minority. In comparison to Jews, the probability of Arabs not RTW was significantly greater. Since income was taken into account in the statistical models, the differences are probably not confounded by socioeconomic status, but rather to cultural and health related parameters, which have been reported elsewhere. In comparison to Jews, Non-Jews in Israel have a 50% significantly higher risk for injury-related hospitalization [40] and are at greater risk for road traffic related mortality [41].

Several explanations may clarify the differences between Arabs and Jews. For example, Arabs may have a greater difficulty in finding a new job after long term unemployment [42], which can contribute to not RTW following an injury. Another factor deterring Arabs from RTW is accessibility to the workplace. There are only a few industrial zones in Arab villages and employment opportunities in Arab communities are limited. Thus, accessibility to place of employment is often difficult and cumbersome [43]. Another explanation for longer out-of-work stay among Arabs may be that living costs are lower in the Arab sector, and thus NII compensation provides enough financial security during a time of unemployment. In addition, in comparison to Jews, Arabs are more likely to have physical labor jobs and hold fewer managerial positions [43] which hinders RTW after an injury.

As detailed, RTW following an injury varies by age and population group, and various factors contribute to the disparities noted between Jews and Arabs in Israel, and between the wealthy and the poor. The outcomes of this study emphasize the importance of promoting RTW programs in general, and specifically in the Arab sector.

Although the odds for longer out of work stay is higher among older adults, the available evidence does not unequivocally support the efficacy of intervention programs promoting RTW for this population group [44], due to fewer productive years prior to retirement.

### Limitations

The inclusion of the specific profession of the injured person in a model that predicts the duration of out of work stay was not possible in this study, since the NII does not include the specific profession (or job description) in its database. While this data would have been useful in predicting RTW by profession, our study was still able to identify specific groups characterized with higher probability of not RTW following an injury. Those defined as at high risk should be further investigated in order to acquire information regarding job description and the intervention should be adjusted to suit each individual.

### Policy implications and recommendations

Multidisciplinary intervention programs focusing on RTW following an injury have proven effective [38, 45–47]. Such programs, which include occupational rehabilitation, social and psychological support as well as monitor the ability to RTW, have shown excellent results in reducing the expenditure of disability allowances in Sweden, Britain and Holland [48].

An Israeli study by Naon et al. [48] examining barriers to RTW following an injury or illness concluded that several steps may contribute to shortening out of work stay, including; 1) providing employers with the responsibility of not only monitoring their employees’ medical conditions, but also adapting the job responsibilities to their medical condition and disabilities; 2) developing clear “sick leave” protocols in an effort to prevent physicians from supplying unnecessary long sick leave permits; and 3) encouraging rehabilitation to begin immediately after illness or injury, in order to prevent the loss of specific skills.

The efficacy of such intervention programs depends on the cooperation of a multidisciplinary body of professionals. Since the NII is in charge of distributing disability funds, it should appoint a coordinator, most probably from the NII, to manage the multidisciplinary team.

As part of the multidisciplinary intervention program, the employer should be involved in the employee rehabilitation process, regardless if the disability was due to an occupational injury. The Ministries of Labor, Social Affairs and Social Services should act for establishing a legislation where employers have a responsibility in the RTW process. It was previously found, that, in cases where workers where not profitable to their employers (especially for low-wage jobs), the employer had no interest in investing in the RTW process [49]. Legislation can enhance the employers’ obligations to accommodate and help their workers RTW, irrespective of the “value” of the employee to the company or business. RTW programs and legislations protect the worker from detrimental or inapprorite tasks which can cause greater damage to the injury, while also enabling the worker to perfom unharmful tasks [50, 51]. This process of occupational rehabilitation should begin as soon as possible after the injury [48]. The government should establish a system of providing positive incentives to employers who participate in a RTW rehabilitation initiative for injured workers and negative incentives to those who choose not to participate.

The Ministry of Health (MoH) should be given several responsibilities regarding RTW intervention programs. The MoH should provide small businesses, which lack an occupational health department or health unit, with professional health care assessment and with a coordinator to ensure an effective RTW process for the injured worker. In addition, the MoH should be accountable for developing culturally adaptive programs.

The Health Maintenance Organizations (HMO’s) should provide the necessary rehabilitation, according the specific needs of each injured worker. This should be done in collaboration with the recommendations of the occupational health care practinioner. Since medical rehabilitation and occupational rehabilitation are related, it is recommended that the two begin simultaneously, immediately after injury [48].

The Workers’ Union should increase awareness among both employers and employees regardomg occupational rehabilitation following an injury. The Unions should also use their power to encourage employers to be active in the RTW process.

The above mentioned professional agencies should collaborate in developing ethnically appropriate interventions, which focus on returning to a productive lifestyle as quickly as possible. In addition, policy makers should use the outcomes and recommendations from this study to identify workers at high risk for long out of work stay following an injury, and to allot an appropriate budget for the resources needed in planning and implementing effective RTW intervention programs.

## Conclusion

This study examined, for the first time in Israel, the time until RTW following occupational and non-occupational injuries. The outcomes identified population groups at high risk for lengthy out of work stay following an injury requiring hospitalization. Policy makers should use the results and recommendations to develop and implement ethnically appropriate intervention programs, with a focus on promoting RTW in order toreduce extensive periods of being out of work.

## Data Availability

Data sharing not applicable to this article as the research center of the National Insurance Institute of Israel does not allow any files, even those without IDs, to be released out of research room of the NII, where all analyses were performed.

## Appendix

**Table 9:** Risk of not RTW within 1 month, 1 year and 2 years expressed in Average Marginal Effect (AME), by demographic and injury characteristics

Demographic and injury characteristics^**a**^	AME (%) [Confidence Interval (95%)]		
	Not RTW within 1 month	Not RTW within 1 year	Not RTW within 2 years^**b**^
**Age**			
21–34	**5.01 [2.79–7.22]**	**−3.87 [− 5.40- -2.34]**	**−5.39 [−6.72- -4.05]**
35–44	**4.27 [1.90–6.63]**	−1.52 [− 3.15–0.10]	**− 3.83 [− 5.25- -2.40]**
45–54	1.76 [− 0.54–4.07]	−0.82 [− 2.40–0.77]	**− 2.99 [− 4.38- -1.61]**
55+ with more than 5 years before retirement age	− 0.97 [− 3.30–1.36]	**− 2.74 [− 4.36- -1.13]**	**−3.15 [− 4.55- -1.75]**
55+ with less than 5 years before retirement age	ref	ref	ref
**Gender** (male vs. female)	**12.33 [11.38–13.29]**	**6.63 [5.88–7.38]**	**3.90 [3.20–4.59]**
**Family Status**			
Married with children	**1.51 [0.16–2.85]**	0.47 [− 0.48–1.43]	**0.91 [0.02–1.80]**
Married without children	ref	ref	ref
Single with children	**4.46 [2.36–6.55]**	**3.42 [1.98–4.86]**	**2.64 [1.29–3.99]**
Single without children	**3.06 [1.68–4.43]**	0.06 [− 0.92–1.04]	0.44 [−0.46–1.35]
**Population Group**			
Other Israelis	ref	ref	ref
Immigrants from FSU	**1.23** **0.04–2.43**	−0.83 [− 1.75–0.08]	**−1.08 [− 1.95- -0.21]**
Ethiopian Immigrants	−0.75 [−3.87–2.37]	1.83 [− 0.27–3.94]	1.47 [− 0.49–3.42]
Israeli Arabs	**8.62 [7.63–9.61]**	**6.08 [5.41–6.75]**	**4.77 [4.14–5.39]**
**Income**			
Higher income (4^th^Quartile)	ref	ref	ref
3^th^Quartile	**16.23 [14.98–17.47]**	**8.12 [6.98–9.25]**	**5.48 [4.36–6.59]**
2^nd^Quartile	**27.58 [26.38–28.79]**	**14.42 [13.33–15.51]**	**9.59 [8.52–10.66]**
Lower income (1^st^Quartile)	**38.89 [37.72–40.06]**	**19.26 [18.18–20.34]**	**13.19 [12.12–14.26]**
**Injury Circumstances**			
Injured not at work	ref	ref	ref
Injured at work - without recognition of NII	**−5.44 [−7.23- -3.64]**	**−1.82 [− 3.23- -0.41]**	−0.97 [−2.28–0.34]
Injured at work - with recognition of NII	**25.09 [24.29–25.89]**	**8.39 [7.77–9.02]**	**5.09 [4.51–5.67]**

^a^The model is adjusted for all variables presented in the Table and in addition to injury mechanism, injury severity and prior-to injury disability

^b^Refers to those injured between 2008–2012

**Table 10:** Risk of not RTW within 1 month, 1 year and 2 years expressed in Average Marginal Effect (AME), by demographic and injury characteristics among aged 21–34

Demographic and injury characteristics^**a**^	AME (%) [Confidence Interval (95%)]		
	Not RTW within 1 month	Not RTWwithin 1 year	Not RTW within 2 years^**b**^
**Gender** (male vs. female)	**12.34 [10.79–13.88]**	**6.88 [5.68–8.07]**	**4.23 [3.13–5.33]**
**Family Status**			
Married with children	**2.89 [0.32–5.46]**	0.15 [− 1.58–1.88]	0.77 [− 0.81–2.35]
Married without children	ref	ref	ref
Single with children	**9.23 [5.02–13.44]**	**4.52 [1.80–7.23]**	**2.94 [0.48–5.40]**
Single without children	**5.37 [2.83–7.92]**	−0.89 [−2.59–0.80]	−0.28 [−1.83–1.27]
**Population Group**			
Other Israelis	ref	ref	ref
Immigrants from FSU	−0.41 [− 2.46–1.65]	−0.47 [−1.99–1.06]	−0.59 [−2.02–0.83]
Ethiopian Immigrants	−0.84 [−5.39–3.71]	2.75 [− 0.16–5.65]	1.99 [− 0.64–4.62]
Israeli Arabs	**7.93 [6.52–9.35]**	**5.78 [4.84–6.71]**	**4.26 [3.41–5.11]**
**Income**			
Higher income (4^th^Quartile)	ref	ref	ref
3^th^Quartile	**11.39 [9.07–13.72]**	**4.37 [2.37–6.37]**	**2.32 [0.47–4.18]**
2^nd^Quartile	**22.62 [20.39–24.84]**	**9.93 [8.01–11.84]**	**5.92 [4.15–7.68]**
Lower income (1^st^Quartile)	**33.50 [31.32–35.67]**	**14.05 [12.15–15.96]**	**8.71 [6.95–10.47]**
**Injury Circumstances**			
Injured not at work	ref	ref	ref
Injured at work - without recognition of NII	**−9.07 [− 11.78- -6.37]**	**−3.93 [− 6.14- -1.72]**	−1.39 [−3.31–0.52]
Injured at work - with recognition of NII	**23.81 [22.49–25.13]**	**7.70 [6.78–8.62]**	**4.70 [3.87–5.54]**

^a^The model is adjusted for all variables presented in the Table and in addition to injury mechanism, injury severity and prior-to injury disability

^b^Refers to those injured between 2008–2012

**Table 11:** Risk of not RTW within 1 month, 1 year and 2 years expressed in Average Marginal Effect (AME), by demographic and injury characteristics among aged 35–44

Demographic and injury characteristics^**a**^	AME (%) [Confidence Interval (95%)]		
	Not RTW within 1 month	Not RTWwithin 1 year	Not RTW within 2 years^**b**^
**Gender** (male vs. female)	**12.92 [10.85–14.99]**	**7.95 [6.22–9.68]**	**4.40 [2.82–5.98]**
**Family Status**			
Married with children	− 0.20 [−4.57–4.16]	0.30 [− 2.88–3.49]	−0.82 [− 3.68–2.03]
Married without children	ref	ref	ref
Single with children	2.10 [− 2.87–7.06]	3.50 [− 0.12–7.12]	1.00 [−2.28–4.28]
Single without children	1.52 [− 3.37–6.40]	− 0.12 [− 3.70–3.46]	− 1.02 [− 4.24–2.20]
**Population Group**			
Other Israelis	ref	ref	ref
Immigrants from FSU	− 0.75 [− 3.29–1.79]	−1.86 [− 3.91–0.20]	**− 2.33 [− 4.32- -0.33]**
Ethiopian Immigrants	− 1.69 [−7.87–4.49]	2.00 [− 2.32–6.31]	2.20 [− 1.69–6.10]
Israeli Arabs	**9.58 [7.53–11.63]**	**5.48 [4.01–6.95]**	**3.87 [2.52–5.23]**
**Income**			
Higher income (4^th^Quartile)	ref	ref	ref
3^th^Quartile	**17.36 [15.06–19.66]**	**10.03 [7.74–12.33]**	**7.38 [4.98–9.77]**
2^nd^Quartile	**28.24 [25.93–30.55]**	**16.80 [14.58–19.02]**	**11.67 [9.32–14.01]**
Lower income (1^st^Quartile)	**39.18 [36.79–41.57]**	**22.18 [19.94–24.42]**	**16.17 [13.82–18.53]**
**Injury Circumstances**			
Injured not at work	ref	ref	ref
Injured at work - without recognition of NII	**− 4.72 [− 8.34- -1.09]**	−1.46 [−4.39–1.47]	−0.74 [−3.44–1.96]
Injured at work - with recognition of NII	**25.15 [23.50–26.80]**	**8.68 [7.27–10.08]**	**4.90 [3.59–6.21]**

^a^The model is adjusted for all variables presented in the Table and in addition to injury mechanism, injury severity and prior-to injury disability

^b^Refers to those injured between 2008–2012

**Table 12:** Risk of not RTW within 1 month, 1 year and 2 years expressed in Average Marginal Effect (AME), by demographic and injury characteristics among aged 45–54

Demographic and injury characteristics^**a**^	AME (%) [Confidence Interval (95%)]		
	Not RTW within 1 month	Not RTWwithin 1 year	Not RTW within 2 years^**b**^
**Gender** (male vs. female)	**13.77 [11.53–16.00]**	**8.00 [6.22–9.78]**	**4.17 [2.52–5.81]**
**Family Status**			
Married with children	1.45 [− 0.90–3.80]	−0.47 [− 2.26–1.33]	0.68 [− 1.02–2.39]
Married without children	ref	ref	ref
Single with children	3.55 [−0.13–7.24]	1.35 [− 1.38–4.08]	**2.80 [0.29–5.31]**
Single without children	0.64 [−2.49–3.77]	0.72 [− 1.62–3.06]	1.03 [− 1.18–3.25]
**Population Group**			
Other Israelis	ref	ref	ref
Immigrants from FSU	2.03 [−0.51–4.57]	**−2.09 [−4.17- -0.02]**	−1.52 [−3.54–0.50]
Ethiopian Immigrants	2.47 [− 5.21–10.15]	−1.27 [−6.89–4.34]	0.14 [−4.70–4.98]
Israeli Arabs	**9.50 [7.06–11.94]**	**6.74 [5.10–8.39]**	**5.65 [4.14–7.17]**
**Income**			
Higher income (4^th^Quartile)	ref	ref	ref
3^th^Quartile	**14.19 [11.74–16.64]**	**9.02 [6.66–11.37]**	**6.77 [4.38–9.17]**
2^nd^Quartile	**27.83 [25.38–30.27]**	**15.82 [13.54–18.10]**	**11.03 [8.68–13.37]**
Lower income (1^st^Quartile)	**41.24 [38.82–43.66]**	**22.25 [20.01–24.48]**	**14.74 [12.42–17.06]**
**Injury Circumstances**			
Injured not at work	ref	ref	ref
Injured at work - without recognition of NII	−2.05 [−6.23–2.14]	1.42 [−1.77–4.61]	0.54 [−2.53–3.61]
Injured at work - with recognition of NII	**25.91 [24.16–27.67]**	**11.10 [9.54–12.66]**	**6.96 [5.47–8.46]**

^a^The model is adjusted for all variables presented in the Table and in addition to injury mechanism, injury severity and prior-to injury disability

^b^Refers to those injured between 2008–2012

**Table 13:** Risk of not RTW within 1 month, 1 year and 2 years expressed in Average Marginal Effect (AME), by demographic and injury characteristics among aged 55+ with more than 5 years before retirement age

Demographic and injury characteristics^**a**^	AME (%) [Confidence Interval (95%)]		
	Not RTW within 1 month	Not RTWwithin 1 year	Not RTW within 2 years^**b**^
**Gender** (male vs. female)	**9.85 [5.56–14.15]**	**5.43 [2.19–8.68]**	**5.12 [1.83–8.40]**
**Family Status**			
Married with children	−3.59 [−7.52–0.34]	−0.83 [−3.58–1.92]	0.66 [−1.85–3.16]
Married without children	ref	ref	ref
Single with children	−0.22 [−8.21–7.77]	1.68 [−3.67–7.02]	−4.30 [−12.01–3.41]
Single without children	−3.41 [− 7.46–0.64]	0.81 [− 2.02–3.64]	1.49 [− 1.18–4.16]
**Population Group**			
Other Israelis	ref	ref	ref
Immigrants from FSU	2.80 [− 0.77–6.36]	−0.42 [−3.03–2.18]	−0.43 [− 2.99–2.12]
Ethiopian Immigrants	− 2.88 [− 17.73–11.97]	− 4.90 [− 17.54–7.75]	–
Israeli Arabs	**8.46 [3.90–13.01]**	**6.69 [3.93–9.45]**	**6.16 [3.60–8.71]**
**Income**			
Higher income (4^th^Quartile)	ref	ref	ref
3^th^Quartile	**15.76 [12.05–19.47]**	**6.63 [3.20–10.06]**	**3.74 [0.28–7.20]**
2^nd^Quartile	**27.23 [23.61–30.85]**	**15.26 [12.09–18.42]**	**9.47 [6.29–12.65]**
Lower income (1^st^Quartile)	**38.68 [34.78–42.59]**	**19.85 [16.59–23.11]**	**15.06 [11.90–18.22]**
**Injury Circumstances**			
Injured not at work	ref	ref	ref
Injured at work - without recognition of NII	**7.20 [0.83–13.57]**	3.29 [−1.35–7.93]	0.45 [−4.37–5.27]
Injured at work - with recognition of NII	**29.30 [26.61–31.99]**	**10.11 [7.82–12.40]**	**6.12 [3.94–8.29]**

^a^The model is adjusted for all variables presented in the Table and in addition to injury mechanism, injury severity and prior-to injury disability

^b^Refers to those injured between 2008–2012

**Table 14:** Risk of not RTW within 1 month, 1 year and 2 years expressed in Average Marginal Effect (AME), by demographic and injury characteristics among aged 55+ with less than 5 years before retirement age

Demographic and injury characteristics^**a**^	AME (%) [Confidence Interval (95%)]		
	Not RTW within 1 month	Not RTW within 1 year	Not RTW within 2 years^**b**^
**Gender** (male vs. female)	**9.60 [6.74–12.46]**	**3.23 [1.13–5.33]**	1.29 [−0.90–3.49]
**Family Status**			
Married with children	1.96 [−5.43–9.34]	−1.02 [− 6.25–4.22]	−1.40 [−6.80–4.00]
Married without children	ref	ref	ref
Single with children	10.80 [− 3.51–25.10]	− 1.92 [− 14.77–10.92]	−3.67 [−17.98–10.64]
Single without children	**5.93 [3.04–8.83]**	1.80 [− 0.31–3.91]	1.23 [− 0.92–3.39]
**Population Group**			
Other Israelis	ref	ref	ref
Immigrants from FSU	3.08 [− 0.20–6.35]	0.08 [−2.34–2.50]	−0.66 [− 3.21–1.90]
Ethiopian Immigrants	−14.48 [− 29.80–0.85]	5.04 [− 3.87–13.95]	4.06 [−5.75–13.87]
Israeli Arabs	**11.68 [6.12–17.23]**	**8.13 [4.75–11.50]**	**8.21 [4.87–11.55]**
**Income**			
Higher income (4^th^Quartile)	ref	ref	ref
3^th^Quartile	**17.95 [13.92–21.98]**	**5.84 [2.27–9.41]**	**5.04 [1.18–8.91]**
2^nd^Quartile	**27.66 [23.83–31.48]**	**11.11 [7.76–14.45]**	**9.47 [5.86–13.08]**
Lower income (1^st^Quartile)	**40.01 [36.74–43.28]**	**17.02 [13.93–20.11]**	**13.99 [10.61–17.37]**
**Injury Circumstances**			
Injured not at work	ref	ref	ref
Injured at work - without recognition of NII	−1.75 [−9.46–5.96]	−2.75 [−8.72–3.22]	−3.81 [−10.50–2.88]
Injured at work - with recognition of NII	**23.13 [20.72–25.55]**	**4.04 [2.09–5.99]**	**2.24 [0.24–4.23]**

^a^The model is adjusted for all variables presented in the Table and in addition to injury mechanism, injury severity and prior-to injury disability

^b^Refers to those injured between 2008–2012
